# Three-year mortality among alcoholic patients after intensive care: a population-based cohort study

**DOI:** 10.1186/cc10603

**Published:** 2012-01-08

**Authors:** Steffen Christensen, Martin B Johansen, Lars Pedersen, Reinhold Jensen, Kim M Larsen, Anders Larsson, Else Tønnesen, Christian F Christiansen, Henrik T Sørensen

**Affiliations:** 1 Department of Clinical Epidemiology, Aarhus University Hospital, Olof Palmes alle 43-45, Aarhus N, 8240, Denmark; 2 Department of Anaesthesiology and Intensive Care, Aarhus University Hospital, Skejby Hospital, Brendstrupgårdsvej 100, Aarhus N, 8240, Denmark; 3 Department of Anaesthesiology and Intensive Care, Aarhus Hospital, Aarhus University Hospital, Nørrebrogade 44, Aarhus C, 8000, Denmark; 4 Department of Anaesthesiology and Intensive Care Medicine, Uppsala University, Uppsala S-751 85, Sweden; 5 Department of Medicine V (Hepatology and Gastroenterology), Aarhus University Hospital, Aarhus Hospital, Aarhus Hospital, Nørrebrogade 44, Aarhus C, 8000, Denmark

## Abstract

**Introduction:**

Alcoholic patients comprise a large proportion of patients in intensive care units (ICUs). However, data are limited on the impact of alcoholism on mortality after intensive care.

**Methods:**

We conducted a cohort study among 16,848 first-time ICU patients between 2001 and 2007 to examine 30-day and 3-year mortality among alcoholic patients. Alcoholic patients with and without complications of alcohol misuse (for example, alcoholic liver disease) were identified from previous hospital contacts for alcoholism-related conditions or redemption of a prescription for alcohol deterrents. Data on medication use, demographics, hospital diagnoses, and comorbidity were obtained from medical databases. We computed 30-day and 3-year mortality and mortality rate ratios (MRRs) by using Cox regression analysis, controlling for covariates.

**Results:**

In total, 1,229 (7.3%) ICU patients were current alcoholics. Among alcoholic patients without complications of alcoholism (*n *= 785, 4.7% of the cohort), 30-day mortality was 15.9% compared with 19.7% among nonalcoholic patients. Compared with nonalcoholic patients, the adjusted 30-day MRR was 1.04 (95% confidence interval (CI), 0.87 to 1.25). Three-year mortality was 36.2% compared with 40.9% among nonalcoholic patients, corresponding to an adjusted 3-year MRR of 1.16 (95% CI, 1.03 to 1.31). For alcoholic patients with complications (*n *= 444, 2.6% of the cohort), 30-day mortality was 33.6%, and 3-year mortality was 64.5%, corresponding to adjusted MRRs, with nonalcoholics as the comparator, of 1.64 (95% CI, 1.38 to 1.95) and 1.67 (95% CI, 1.48 to 1.90), respectively.

**Conclusions:**

Alcoholic ICU patients with chronic complications of alcoholism have substantially increased 30-day and 3-year mortality. In contrast, alcoholics without complications have no increased 30-day and only slightly increased 3- year mortality.

## Introduction

Alcoholism is a major public health problem [1]. An estimated 15- to 20-million people in the United States are chronic alcohol abusers, and more than 100,000 deaths in the United States are ascribed annually to alcoholism [1]. Alcoholic patients hospitalized with pneumonia or trauma or undergoing major elective surgery are at increased risk of serious complications, such as acute respiratory distress syndrome and sepsis [2-10]. The prevalence of patients in intensive care units (ICUs) with definite alcoholism was reported to be 12% in a 1988 Danish study of 216 ICU patients [11], and as many 28% of ICU patients admitted to a UK inner-city hospital were admitted with alcohol-related complications [6].

No randomized controlled study examined the effect of alcoholism on ICU prognosis [12]. Interpretation of the few and inconsistent observational studies on the short-term prognosis of alcoholic patients in general ICUs is complicated by inclusion of highly selected study populations, such as trauma patients and patients undergoing major surgery, or by inclusion of ICU patients from hospitals primarily treating mentally ill and alcohol/drug abusing patients [3,13,14]. Relatively small study populations and lack of control for potential confounding factors, including comorbidity, further complicate the interpretation of the studies [2,3,6,11,13-15]. Moreover, limited data exist on whether any increased mortality is related to alcoholism itself or to chronic complications of excessive alcohol abuse, such as liver cirrhosis [13]. To our knowledge, no data exist on the mortality of alcoholic ICU patients beyond 30 days after ICU admission.

Data on the prognosis of alcoholic ICU patients are important for understanding their clinical course and thus for preventing complications in these high-risk patients. We conducted a cohort study encompassing more than 16,000 ICU patients to examine the association between alcoholism (with and without chronic complications of alcoholism) and mortality after intensive care.

## Materials and methods

### Setting and study population

The study cohort included ICU patients admitted between January 1, 2001, and December 31, 2007, to three ICUs (at Aalborg, Skejby, and Aarhus Hospitals) within the Aarhus University Hospital Collaboration, Aarhus, Denmark. Prospectively collected patient data were merged electronically into a research database known as the Aarhus University Intensive Care Cohort (AUICC) database. All ICUs in the Aarhus University Hospital Collaboration are highly specialized multidisciplinary tertiary teaching units serving as both primary and referral ICUs. Dedicated ICU teams are responsible for the care of all ICU patients. Together, the ICUs cover all major medical specialities. The nurse-to-patient ratio is 1:1.

We included only first-time ICU admissions during the study period and excluded patients transferred to the ICUs for planned postoperative observation of less than 24 hours. Also, only ICU patients older than 15 years were included. This yielded a study population of 16,848 first-time adult ICU patients. The AUICC database contains data on all ICU admissions during the study period, including date of ICU admission and discharge, use of mechanical ventilation, and renal replacement therapy.

### Identification of alcoholic patients

We obtained data on all previous hospitalizations with complications of alcoholism (for example, alcoholic liver disease) through the Danish National Registry of Patients (DNRP) (for details, see Additional file 1). The DNRP contains key information on all inpatient hospitalizations at nonpsychiatric hospitals in Denmark since 1977 and on all outpatient clinic and emergency department visits since 1995 [16]. Data include patients' civil registration numbers, admission and discharge dates, department providing care, up to 20 surgical procedures, and up to 20 discharge diagnoses, coded according to the *International Classification of Diseases*, 8th edition (ICD-8) until the end of 1993, and 10th edition (ICD-10) thereafter.

We obtained data on the use of alcohol deterrents (primarily disulfiram) and other prescription medications through a prescription database with complete coverage in the study region since 1998 [17]. The database contains key information on prescriptions for all reimbursed drugs dispensed from every pharmacy in the region. Danish guidelines specify disulfiram as the primary treatment for alcohol abuse.

Based on the complete hospital and prescription history of patients in the study cohort, we defined alcoholic patients as those with (a) at least one redeemed prescription for an alcohol deterrent within 1 year preceding ICU admission, and/or (b) at least one hospital or outpatient clinic/emergency department visit with a diagnosis of an alcoholism-related disease registered within 1 year of ICU admission (for details on ATC and ICD codes, see Additional file 1). Nonalcoholic ICU patients were defined as those without redeemed prescriptions for an alcohol deterrent and without any alcoholism-related diagnosis. The 1-year time window reduced the risk of incorrectly classifying former alcoholic patients as current alcoholics.

### Subcohorts of alcoholic ICU patients

We further categorized alcoholic patients into two subcohorts: (a) patients with complications of alcoholism (that is, alcoholic psychosis, alcoholic pancreatitis, alcoholic liver diseases, alcoholic gastritis, alcoholic neuropathy, alcoholic myopathy, alcoholic dementia, and alcoholic cardiomyopathy); and (b) patients without complications of alcoholism (that is, those with alcohol dependence, alcohol abuse, and/or use of alcohol deterrents). Details on categorization based on diagnosis and prescription data are provided in the Additional file 1. We further identified the subgroup of patients in whom the alcoholic complication was liver cirrhosis.

In a sensitivity analysis, we categorized alcoholic patients based on their entire hospital and prescription history both since 1977 and since 1998.

### Covariates

To control for the impact of comorbid diseases on mortality, we used the summary measure of comorbidities developed by Charlson *et al. *(the Charlson Comorbidity Index (CCI) score), by using a coding scheme validated for use with hospital discharge data [18-20]. The Charlson index includes 19 groups of major chronic diseases, including ischemic heart disease, cancer, diabetes, chronic lung diseases, and chronic renal failure. We obtained data on the complete post-1977 hospital history preceding the date of ICU admission from the DNRP to compute a CCI score for each patient. We defined three levels of comorbidity: low (score of 0); medium (score of 1 to 2); and high (score of > 2). We did not include alcoholism-related diseases in the CCI, as they constituted our exposure variable.

We also used the DNRP to obtain data on the primary diagnoses associated with the hospitalization during which patients were admitted to the ICU, and on all surgical procedures performed on study patients within 30 days before ICU admission. To measure social status, we obtained data from the Danish Civil Registration System (CRS) on place of residence (that is, urban versus rural) and marital status at the time of ICU admission [21].

### Mortality data

To obtain information on death or migration, we linked the study cohort to the Danish CRS, which has maintained a database on vital status (dead, alive) and exact date of death for the entire Danish population, updated daily, since 1968 [21,22].

### Statistical analysis

Follow-up extended from the date of first-time ICU admission until death or migration or until 3 years after ICU admission, whichever came first. We computed Kaplan-Meier life-table estimates for 30-day and 3-year mortality for the main variables (that is, alcoholism with and without complications, age group (15 to 44, 45 to 59, 61 to 74, and 75+ years), gender, level of comorbidity, primary hospital registry diagnosis (infectious disease, cancer, diabetes, cardiovascular disease, respiratory disease, gastrointestinal/liver disease, trauma/poisoning, and other), department providing care (medical/surgical), surgical procedures, type of admission (emergency/planned), and marital status (married, divorced, never married, widow(er), unknown).

We used the Cox regression analysis to compare 30-day and 3-year mortality among alcoholic patients with and without alcoholism-related complications with those of nonalcoholic patients. We estimated mortality rate ratios (MRRs) with 95% confidence intervals (CIs), controlling for the covariates listed earlier and allowing a time-varying effect when relevant. Because age may influence the impact of alcoholism on mortality, we repeated the analysis stratified by age group. Some indications exist that alcoholism is associated with increased mortality after major surgery, and we therefore also did an analysis stratified by department (medical/surgical). Liver cirrhosis is associated with increased mortality after ICU admission, and we therefore also did a stratified analysis by liver cirrhosis among patients with alcoholic complications. Finally, we repeated the regression analysis after identifying and categorizing alcoholic patients on the basis of their entire available prescription and hospital history.

The assumptions of proportional hazards in the Cox model were assessed graphically and found appropriate for each follow-up period.

All analyses were performed by using SAS version 9.2 (SAS Institute Inc, Cary, NC)

All data were obtained from Danish registries, which are generally available to researchers, and their use does not require informed consent. The study was approved by the Danish Data Protection Agency and the Aarhus University Hospital Registry Board.

## Results

We identified 16,848 first-time ICU patients, of whom 1,229 (7.3%) were current alcoholics. Among these patients, 785 (4.7%) had a prescription for an alcohol deterrent or a history of alcoholism-related hospitalization without complications of alcoholism within 1 year before ICU admission; 444 (2.6%) had a history of alcoholism-related hospitalization with complications of alcoholism within 1 year before ICU admission (Table 1).

**Table 1:** Baseline characteristics of alcoholic patients with and without complications and nonalcoholic patients admitted to ICUs within the Aarhus University Hospital Network, Denmark, 2001 to 2007

	Nonalcoholic patients		Alcoholic patients with complications		Alcoholic patients without complications	
	**(*n*)**	**(%)**	**(*n*)**	**(%)**	**(*n*)**	**(%)**

**Overall**	15,619	92.7	444	2.6	785	4.7

**Age (years)**						
15-44	3,559	22.8	86	19.4	253	32.2
45-59	3,353	21.5	226	50.9	340	43.3
60-74	5,400	34.6	123	27.7	166	21.2
75+	3,307	21.2	9	2.0	26	3.3
**Gender**						
Female	6,530	41.8	130	29.3	239	30.5
Male	9,089	58.2	314	70.7	546	69.6
**Comorbidity score^a^**						
Low	6,481	41.5	124	27.9	395	50.3
Medium	5,628	36.0	137	30.9	288	36.7
High	3,510	22.5	183	41.2	102	13.0
**Comorbidity**						
Ischemic heart disease	1,682	10.8	17	3.8	18	2.3
Congestive heart failure	1,564	10.0	27	6.1	30	3.8
Peripheral vascular disease	1,550	9.9	27	6.1	38	4.8
Cerebrovascular disease	1,915	12.3	46	10.4	82	10.5
COPD	2,331	14.9	69	15.5	114	14.5
Diabetes	1,389	8.9	67	15.1	56	7.1
Cancer	2,831	18.1	41	9.2	73	9.3
**Marital status**						
Married	7,609	48.7	154	34.7	225	28.7
Divorced	3,165	20.3	109	24.6	265	33.8
Widow(er)	1,777	11.4	134	30.2	233	29.7
Never married	2,402	15.4	29	6.5	45	5.7
Unknown	666	4.3	18	4.1	17	2.2

**^a^**Level of Charlson Comorbidity Index (see text for details). COPD, chronic obstructive pulmonary disease.

### Descriptive data

Alcoholic patients were more likely to be men and were younger (26.4% were older than 60 years) than were nonalcoholic patients (55.7% were older than 60 years). Alcoholic patients with complications of alcoholism had higher comorbidity scores than did other patients. For the hospitalization that required ICU admission, gastrointestinal diseases were more frequent among alcoholics than among nonalcoholics, whereas nonalcoholics were more often hospitalized with cardiovascular diseases and cancer (Table 2). Fifty-nine percent of alcoholic patients were transferred to the ICU from medical departments, compared with 40% of nonalcoholic patients. Slightly fewer alcoholic patients than nonalcoholic patients were treated with mechanical ventilation and renal replacement therapy. A total of 379 (51.7%) alcoholic patients with complications were registered with a diagnosis of liver cirrhosis.

**Table 2:** Characteristics associated with the current hospitalization of ICU patients, Aarhus University Hospital Network, Denmark, 2001 to 2007

	Nonalcoholic patients		Alcoholic patients with complications		Alcoholic patients without complications	
	***n***	**%**	***n***	**%**	***n***	**%**

Planned/emergency hospital admission						
Planned	3,665	23.5	44	9.9	59	7.5
Emergency	11,954	76.5	400	90.1	726	92.4

Primary diagnosis						
Infectious disease	392	2.5	15	3.4	33	4.2
Cancer	2,208	14.1	15	3.4	42	5.4
Diabetes	200	1.3	6	1.4	13	1.7
Cardiovascular disease	4,310	27.6	93	21.0	84	10.7
Respiratory disease	1,443	9.2	23	5.2	66	8.4
Gastrointestinal disease	1,419	9.1	189	42.6	99	12.6
Trauma/poisoning	3,255	20.8	44	9.9	275	35.0
Other	2,393	15.3	59	13.3	173	22.0

Surgical procedures						
No surgery	7,830	50.1	339	76.4	555	70.7
Vascular	2,647	17.0	15	3.4	37	4.7
Abdominal	1,342	8.6	36	8.1	40	5.1
Orthopedic	881	5.6	9	2.0	15	1.9
Thoracic	743	4.8	29	6.5	48	6.1
Central nervous System	1,452	9.3	8	1.8	54	6.9
Other	724	4.6	8	1.8	36	4.6

Department						
Surgical	9,376	60.0	181	40.8	326	41.5
Medical	6,243	40.0	263	59.2	459	58.5

Mechanical ventilation						
Yes	6,692	42.9	172	38.7	308	39.2

Renal replacement therapy						
Yes	1,160	7.4	38	8.6	46	5.9

### 30-day mortality

**Figure 1:**
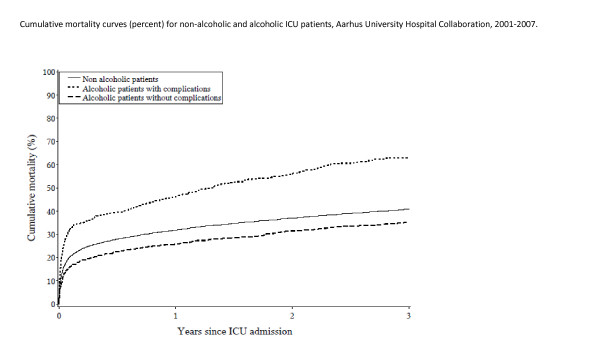
**Cumulative mortality curves (percentages) for nonalcoholic and alcoholic ICU patients, Aarhus University Hospital Collaboration, 2001 to 2007**.

Patients with alcoholism-related complications were at higher risk of death than were nonalcoholics throughout the follow-up period (Figure 1). Thirty-day mortality among alcoholic patients without complications was 15.9%, increasing to 33.6% for alcoholic patients with complications, compared with 19.7% for nonalcoholic patients (Table 3). The corresponding adjusted 30-day MRRs for alcoholic patients with and without complications were 1.64 (95% CI, 1.38 to 1.95) and 1.04 (95% CI, 0.87 to 1.25), respectively, compared with nonalcoholic patients.

**Table 3:** The 30-day and 3-year mortality and corresponding crude and adjusted mortality rate ratios for alcoholic and nonalcoholic ICU patients

	30-day mortality			3-year mortality		
	**Mortality % (95% CI)**	**Crude** **MRR** **(95% CI)**	**Adjusted MRR** **(95% CI) Ω**	**Mortality % (95% CI)**	**Crude MRR** **(95% CI)**	**Adjusted MRR** **(95% CI) Ω**

**Overall**						
Nonalcoholic patients	19.7 (19.1-20.3)	1.0 (reference)	1.0 (reference)	40.9 (40.1-41.6)	1.0 (reference)	1.0(reference)
Alcoholic patients, with complications	33.6 (29.4-38.2)	1.83 (1.55-2.16)	1.64 (1.38-1.95)	64.5 (60.0-69.0)	1.89 (1.68-2.13)	1.67 (1.48-1.90)
Alcoholic patients, no complications	15.9 (13.5-18.7)	0.79 (0.66-0.94)	1.04 (0.87-1.25)	36.2 (32.9-39.7)	0.84 (0.75-0.95)	1.16 (1.03-1.31)

Ω Adjusted by Cox proportional hazards analysis for age group, gender, department providing care (medical/surgical), primary diagnosis, surgery, Charlson index score, emergency/planned admission, and marital status.

### 3-year mortality

Among nonalcoholic patients, 3-year mortality was 40.9%, compared with 36.2% among alcoholic patients without complications of alcoholism and 64.5% among patients with complications. Adjusted MRRs for alcoholic patients with and without complications were 1.67 (95% CI, 1.48 to 1.90) and 1.16 (95% CI, 1.03 to 1.31), respectively, compared with nonalcoholic patients. The single most important confounding factor driving the relative risk from the unadjusted MRR of 0.84 to the adjusted MR of 1.16 was the age differences between alcoholic and nonalcoholic patients (as seen in Table 1).

For alcoholic patients younger than age 45 with complications of alcoholism, 30-day and 3-year MRRs were substantially increased (adjusted 30-day MRR, 3.26 (95% CI, 1.99 to 5.33), and adjusted 3-year MRR, 3.19 (95% CI, 2.27 to 4.48)). The corresponding adjusted 30-day MRR for patients aged 75+ years was 1.07 (95% CI, 0.44 to 2.58), and the 3-year adjusted MRR was 1.11 (95% CI, 0.55 to 2.22). Among medical ICU patients, the adjusted 30-day MRR for those with complications of alcoholism was 1.57 (95% CI, 1.25 to 1.97), and for those without complications, it was 0.78 (95% CI, 0.60 to 1.02). For surgical ICU patients, the corresponding MRRs were 1.53 (95% CI, 1.19 to 1.97) and 1.32 (95% CI, 0.97 to 1.78), respectively. The increased 30-day mortality among alcoholic patients with complications was restricted to those with liver cirrhosis (with liver cirrhosis, adjusted MRR was 1.92 (95% CI, 1.59 to 2.33), and without liver cirrhosis, adjusted MRR was 0.98 (95% CI, 0.66 to 1.45)). Among alcoholics with complications, 3-year mortality for alcoholics with liver cirrhosis was 73.4%, with 46.9% for alcoholics with noncirrhotic complications. When compared with those of nonalcoholics, adjusted MRRs were 1.89 (95% CI, 1.64 to 2.18) for alcoholic patients with cirrhosis and 1.25 (95% CI, 0.98 to 1.58) for alcoholics with noncirrhotic complications.

When identification and categorization were based on patients' entire prescription and hospital histories, MRRs for alcoholic patients with complications remained elevated, although at a slightly lower level (the 30-day adjusted MRR among alcoholics with complications was 1.44 (95% CI, 1.24 to 1.67), and the 3-year adjusted MRR was 1.55 (95% CI, 1.39 to 1.71)).

## Discussion

In this large cohort study, we found that alcoholic patients admitted to ICUs had substantially increased short- and long-term mortality if they had complications of alcoholism such as alcoholic liver disease. The increased mortality was most pronounced in alcoholics with liver cirrhosis. We found no increased 30-day mortality among alcoholics without complications and only slightly increased 3-year mortality.

Our data extend previous research findings on alcoholism and ICU outcomes in several ways, including complete long-term follow-up and better control for confounding, in particular, from comorbidity. A Danish cohort study published almost 20 years ago found significantly increased short-term mortality of 50% among alcohol abusers compared with 26% among nonabusers; however, the study did not control for potential confounders and did not report separate mortality estimates for those with and without complications of alcoholism [11].

In line with our findings, a 1996 German observational study reported increased short-term mortality (7% versus 0) among chronic alcoholic patients admitted to a surgical ICU after major upper gastrointestinal surgery [8]. A 2007 U.S. observational study based on administrative data from "safety net" hospitals (primarily serving special populations, including the mentally ill and alcohol abusers) found that alcohol dependence among ICU patients in this setting was associated with significantly increased in-hospital mortality [13]. Also in accordance with our findings, alcohol dependence complicated by liver diseases was associated with higher in-hospital mortality rates than was alcohol dependence without such complications. This suggests that any increased mortality among alcoholic ICU patients may be caused, not by alcoholism itself but by the complications related to alcohol abuse. This is further supported by an Austrian cohort study reporting similar in-hospital mortality rates for alcoholics and nonalcoholic liver cirrhosis patients admitted to a mixed medical/surgical ICU [14]. In contrast, a Finnish cohort study reported similar in-hospital mortality among patients admitted to ICUs with and without alcohol-related conditions; however, use of alcohol-related ICU admissions as the main exposure in contrast to chronic alcoholism used in the present study complicates further comparisons between the studies [15].

No previous study has presented separate mortality data for young and elderly alcoholic ICU patients. In the present study, the increased mortality appeared to be most pronounced among young alcoholic ICU patients. Why younger alcoholic patients have particularly high relative mortality in the years after ICU admission is not entirely clear. However, young alcoholics may be more-severe abusers, with an overall poorer prognosis than older alcoholics, when compared with nonalcoholic ICU patients in their age group. We lacked data to examine this further.

The longitudinal population-based medical databases used in our research permitted examination, for the first time, of long-term mortality among alcoholic ICU patients, with complete follow-up. Long-term mortality is more complex to interpret than 30-day mortality, when most deaths are directly linked to ICU admission. With increasing length of follow-up, patients may be more likely to suffer progression of their alcoholic complications and other lifestyle-related diseases, and to receive other treatments that may affect their prognosis. In particular, the modest increase in mortality among alcoholic patients with complications warrants careful interpretation. Still, from a public health perspective, data on long-term mortality among alcoholic ICU patients are important for understanding these patients' clinical course.

Accurate identification of alcoholic patients in ICUs is difficult and often prone to selection and information bias when based on interviews with the patients or their relatives. We identified alcoholic patients based on previous hospitalizations with alcohol-related disorders or by redemption of prescriptions for alcohol deterrents within 1 year before ICU admission [7]. This information was prospectively collected, independent of the current study, which largely eliminated the risk of selection and information biases. Co-payment requirements for alcohol deterrents most likely increase the specificity of information on their use.

Misclassification of alcoholic patients because of underreporting of alcoholism-related diagnoses in the databases may have affected our results. In the current study, 7.3% of all patients were classified as alcoholics. This is slightly lower than previously reported in a Danish ICU study (12%) and substantially lower than reported from an inner-city hospital in the United Kingdom (28%) [6,11]. These differences may be explained by differences in the definition of alcoholism and methods of identifying alcoholics in the ICUs (that is, register data versus interviews), but may also represent differences in case-mix among study cohorts. Such differences may explain at least part of the differences between studies on the prognosis of alcoholic ICU patients.

Use of previous hospitalizations and prescription drug use to identify alcoholic patients may have led to inclusion of only those alcoholic patients with the most severe alcohol abuse. This may have inflated our absolute mortality estimates. Any misclassification of alcoholic patients was most likely independent of mortality and therefore would lead to an underestimation of the relative mortality estimates. However, we found no increased mortality among alcoholics without complications of alcoholism, and identifying alcoholic patients based on their entire prescription history versus within 1 year before ICU admission had no major impact on relative mortality estimates. We therefore believe that any influence from such factors is most likely minor. Unfortunately, the registries used for the present study did not contain data on actual alcohol consumption. However, even in studies based on primary data collection, it is difficult to obtain valid data on actual alcohol consumption from ICU patients: alcoholic patients may not be entirely accurate about their actual alcohol consumption, or may be sedated or unconscious for other reasons.

Despite the free tax-supported Danish public health care system, it is possible that lack of ICU capacity leads to some reluctance to admit socially marginalized end-stage alcoholic patients. This may have influenced our results. We may only speculate on the reason for the minor difference in mechanical ventilation between alcoholics and nonalcoholics. However, alcoholics are more likely than nonalcoholics to be admitted to the ICU because of nonrespiratory complications, such as upper GI bleeding, low Glasgow Coma Scale score, or severe electrolyte derangement. Thus differences in mechanical ventilation do not necessarily reflect differences in severity of illness. We lacked clinical data on severity of illness at ICU admission (for example, APACHE or SAPS scores). However, such scores were developed for clinical prediction rather than to control for confounding in etiologic research [23]. Also, chronic alcohol abuse induces immune dysfunction and failure of a number of organ systems included in severity-of-illness scores [24]. Thus the level of APACHE or SAPS scores may be considered an effect of alcoholism rather than a potential confounding factor [25]. Control for severity of illness potentially could bias the relative-risk estimates toward the null. In the present study, we controlled individually for a wide range of covariates, such as age and comorbidity. When used in a prediction model, these factors have been shown to predict ICU mortality as well as APACHE and SAPS scores [26]. We lacked data on lifestyle factors such as smoking and obesity, which may have influenced our results, but controlled for a wide range of consequences of these lifestyle factors, such as chronic lung diseases, ischemic heart diseases, cancer, diabetes, and chronic renal failure.

For several reasons, alcoholism may be associated with increased mortality after ICU admission [10]. Physiological changes associated with alcoholism include immune suppression [27,28], acute renal failure, alteration of hemostatic functions [29] and stress responses [30], poor wound healing [31], and congestive heart failure [32,33]. Critically ill alcoholic patients often have complications requiring urgent and specific treatments, such as alcoholic ketoacidosis, lactate acidosis, severe electrolyte disturbances, or delirium from alcohol withdrawal [24].

In clinical settings, early recognition and treatment of these alcoholism-specific conditions is complicated by difficulty identifying alcoholic patients. Subsequent delayed treatment may increase mortality [34]. The current study indicates that special attention should be paid to ICU patients with chronic complications of alcoholism.

## Conclusion

Alcoholic patients with chronic complications of alcoholism have substantially increased short-term and long-term mortality after ICU admission. Thirty-day mortality was not increased among alcoholics without complications, and 3-year mortality was only slightly increased.

## Key messages

• Alcoholism and alcoholism-related complications are common in intensive care unit patients

• Alcoholic patients with chronic complications of alcohol abuse have substantial increased 30-day and 3-year mortality

• In alcoholic patients without alcoholism-related complications, 30-day mortality is not increased, and 3-year mortality is only slightly increased

## Abbreviations

AUICC: Aarhus University Intensive Care Cohort; CCI: Charlson Comorbidity Index; CI: confidence interval; CRS: Civil Registration System; DNRP: Danish National Registry of Patients; ICD: International Classification of Diseases; ICU: intensive care unit; MRR: mortality rate ratio.

## Competing interests

The authors declare that they have no competing interests.

## Authors' contributions

SC, AL, ET, RJ, KML, and HTS conceived the study idea. SC, MBJ, CFC, and HTS designed the study. LAP, HTS, RJ, and KML collected the data. MBJ, SC, CFC, and LP analyzed the data. All authors interpreted the findings. SC, CFC, and AL reviewed the literature. SC wrote the first draft, and all authors edited the manuscript and approved the final version.

## Supplementary Material

Additional file 1 **A. International Classification of Diseases (ICD)-10 Codes and Anatomical Therapeutic Chemical (ATC) prescription codes used for identification and classification of alcoholic patients**. International Classification of Diseases (ICD)-10 codes and Anatomical Therapeutic Chemical (ATC) prescription codes used for identification and classification of alcoholic patients with and without alcohol-related complications and liver cirrhosis. **B. Charlson Comorbidity Index and comorbidity groups**. ICD-8 and ICD-10 codes used to identify diagnosis included in the Charlson Comorbidity Index and a description of the groups of chronic diseases. Click here for file

## References

[B1] O'Connor PG Schottenfeld RS Patients with alcohol problems N Engl J Med 1998 338 592 602 10.1056/NEJM199802263380907 9475768

[B2] Gacouin A Legay F Camus C Volatron AC Barbarot N Donnio PY Thomas R Le TY At-risk drinkers are at higher risk to acquire a bacterial infection during an intensive care unit stay than abstinent or moderate drinkers Crit Care Med 2008 36 1735 41 10.1097/CCM.0b013e318174dd75 18520640

[B3] Gado-Rodriguez M Gomez-Ortega A Mariscal-Ortiz M Palma-Perez S Sillero-Arenas M Alcohol drinking as a predictor of intensive care and hospital mortality in general surgery: a prospective study Addiction 2003 98 611 6 10.1046/j.1360-0443.2003.00353.x 12751978

[B4] Marik P Mohedin B Alcohol-related admissions to an inner city hospital intensive care unit Alcohol Alcohol 1996 31 393 6 8879288 10.1093/oxfordjournals.alcalc.a008168

[B5] Maxson PM Schultz KL Berge KH Lange CM Schroeder DR Rummans TA Probable alcohol abuse or dependence: a risk factor for intensive-care readmission in patients undergoing elective vascular and thoracic surgical procedures; Mayo Perioperative Outcomes Group Mayo Clin Proc 1999 74 448 53 10319073

[B6] Mostafa SM Murthy BV Alcohol-associated admissions to an adult intensive care unit: an audit Eur J Anaesthesiol 2002 19 193 6 12071239 10.1017/s0265021502000340

[B7] Saitz R Ghali WA Moskowitz MA The impact of alcohol-related diagnoses on pneumonia outcomes Arch Intern Med 1997 157 1446 52 10.1001/archinte.1997.00440340078008 9224223

[B8] Spies CD Nordmann A Brummer G Marks C Conrad C Berger G Runkel N Neumann T Müller C Rommelspacher H Specht M Hannemann L Striebel HW Schaffartzik W Intensive care unit stay is prolonged in chronic alcoholic men following tumor resection of the upper digestive tract Acta Anaesthesiol Scand 1996 40 649 56 10.1111/j.1399-6576.1996.tb04505.x 8836256

[B9] Spies CD Neuner B Neumann T Blum S Müller C Rommelspacher H Rieger A Sanft C Specht M Hannemann L Striebel HW Schaffartzik W Intercurrent complications in chronic alcoholic men admitted to the intensive care unit following trauma Intensive Care Med 1996 22 286 93 10.1007/BF01700448 8708164

[B10] Tonnesen H Kehlet H Preoperative alcoholism and postoperative morbidity Br J Surg 1999 86 869 74 10.1046/j.1365-2168.1999.01181.x 10417555

[B11] Jensen NH Dragsted L Christensen JK Jorgensen JC Qvist J Severity of illness and outcome of treatment in alcoholic patients in the intensive care unit Intensive Care Med 1988 15 19 22 3230195 10.1007/BF00255630

[B12] Sørensen HT Lash TL Rothman KJ Beyond randomized controlled trials: a critical comparison of trials with nonrandomized studies Hepatology 2006 44 1075 82 10.1002/hep.21404 17058242

[B13] O'Brien JM Jr Lu B Ali NA Martin GS Aberegg SK Marsh CB Lemeshow S Douglas IS Alcohol dependence is independently associated with sepsis, septic shock, and hospital mortality among adult intensive care unit patients Crit Care Med 2007 35 345 50 10.1097/01.CCM.0000254340.91644.B2 17205003

[B14] Zauner C Schneeweiss B Kranz A Klos H Gendo A Ratheiser K Lenz K Kramer L Madl C Heavy chronic alcohol abuse has no additional adverse effect on the function of extrahepatic organs and ICU mortality in patients with liver cirrhosis Wien Klin Wochenschr 1999 111 810 4 10568012

[B15] Uusaro A Parviainen I Tenhunen JJ Ruokonen E The proportion of intensive care unit admissions related to alcohol use: a prospective cohort study Acta Anaesthesiol Scand 2005 49 1236 40 10.1111/j.1399-6576.2005.00839.x 16146458

[B16] Ehrenstein V Antonsen S Pedersen L Existing data sources for clinical epidemiology: Aarhus University Prescription Database Clin Epidemiol 2010 2 273 9 21152254 10.2147/CLEP.S13458 PMC2998817

[B17] Andersen TF Madsen M Jorgensen J Mellemkjoer L Olsen JH The Danish National Hospital Register: a valuable source of data for modern health sciences Dan Med Bull 1999 46 263 8 10421985

[B18] Charlson ME Pompei P Ales KL MacKenzie CR A new method of classifying prognostic comorbidity in longitudinal studies: development and validation J Chronic Dis 1987 40 373 83 10.1016/0021-9681(87)90171-8 3558716

[B19] Thygesen SK Christiansen CF Christensen S Lash TL Sørensen HT The predictive value of ICD-10 diagnostic coding used to assess Charlson comorbidity index conditions in the population-based Danish National Registry of Patients BMC Med Res Methodol 2011 11 83 10.1186/1471-2288-11-83 21619668 PMC3125388

[B20] Deyo RA Cherkin DC Ciol MA Adapting a clinical comorbidity index for use with ICD-9-CM administrative databases J Clin Epidemiol 1992 45 613 9 10.1016/0895-4356(92)90133-8 1607900

[B21] Pedersen CB Gøtzsche H Møller JO Mortensen PB The Danish Civil Registration System: a cohort of eight million persons Dan Med Bull 2006 3 441 9 17150149

[B22] Frank L Epidemiology: when an entire country is a cohort Science 2000 287 2398 9 10.1126/science.287.5462.2398 10766613

[B23] Le Gall Jr Lemeshow S Saulnier F A new Simplified Acute Physiology Score (SAPS II) based on a European/North American multicenter study JAMA 1993 270 2957 63 10.1001/jama.1993.03510240069035 8254858

[B24] Moss M Burnham EL Alcohol abuse in the critically ill patient Lancet 2007 368 2231 42 17189035 10.1016/S0140-6736(06)69490-7

[B25] Rothman KJ Epidemiology, an introduction 2002 1 New York: Oxford University Press

[B26] Christensen S Johansen MB Christiansen CF Jensen R Lemeshow S Comparison of Charlson comorbidity index with SAPS and APACHE scores for prediction of mortality following intensive care Clin Epidemiol 2011 3 203 11 21750629 10.2147/CLEP.S20247 PMC3130905

[B27] Chiappelli F Kung M Lee P Pham L Manfrini E Villanueva P Alcohol modulation of human normal T-cell activation, maturation, and migration Alcohol Clin Exp Res 1995 19 539 44 10.1111/j.1530-0277.1995.tb01545.x 7573771

[B28] Jerrells TR Immunodeficiency associated with ethanol abuse Adv Exp Med Biol 1991 288 229 36 1950734 10.1007/978-1-4684-5925-8_26

[B29] Rubin R Effect of ethanol on platelet function Alcohol Clin Exp Res 1999 23 1114 8 10.1111/j.1530-0277.1999.tb04234.x 10397300

[B30] Groote VR Meinders AE On the mechanism of alcohol-induced pseudo-Cushing's syndrome Endocr Rev 1996 17 262 8 8771359 10.1210/edrv-17-3-262

[B31] Rantala A Lehtonen OP Niinikoski J Alcohol abuse: a risk factor for surgical wound infections? Am J Infect Control 1997 25 381 6 10.1016/S0196-6553(97)90082-1 9343620

[B32] Patel VB Why HJ Richardson PJ Preedy VR The effects of alcohol on the heart Adverse Drug React Toxicol Rev 1997 16 15 43 9192055

[B33] Thomas AP Rozanski DJ Renard DC Rubin E Effects of ethanol on the contractile function of the heart: a review Alcohol Clin Exp Res 1994 18 121 31 10.1111/j.1530-0277.1994.tb00891.x 8198208

[B34] Spies C Tonnesen H Andreasson S Helander A Conigrave K Perioperative morbidity and mortality in chronic alcoholic patients Alcohol Clin Exp Res 2001 25 164S 70S 10.1111/j.1530-0277.2001.tb02392.x 11391067

