# Supplementary material for: Three-year mortality among alcoholic patients after intensive care: a population-based cohort study
Source: Crit Care. 2012 Jan 8;16(1):R5. doi: 10.1186/cc10603 (PMC3396230; doi:10.1186/cc10603)
Supplement: Additional file 1 — A. International Classification of Diseases (ICD)-10 Codes and Anatomical Therapeutic Chemical (ATC) prescription codes used for identification and classification of alcoholic patients. International Classification of Diseases (ICD)-10 codes and Anatomical Therapeutic Chemical (ATC) prescription codes used for identification and classification of alcoholic patients with and without alcohol-related complications and liver cirrhosis. B. Charlson Comorbidity Index and comorbidity groups. ICD-8 and ICD-10 codes used to identify diagnosis included in the Charlson Comorbidity Index and a description of the groups of chronic diseases. [file cc10603-S1.DOC]

**Appendix**

**A. International Classification of Diseases (ICD)-10 codes and Anatomical Therapeutic Chemical (ATC) prescription codes used for identification and classification of alcoholic patients**

1. Alcoholic patients with alcohol-related complications

ICD-10: F10.7-F10.9 (alcoholic psychosis), K86.0 (alcoholic pancreatitis), K70 (alcoholic liver diseases), K29.2 (alcoholic gastritis), G62.1 (alcoholic neuropathy), G72.1 (alcoholic myopathy), G31.2.(alcoholic dementia), and I42.6 (alcoholic cardiomyopathy)

2. Alcoholic patients without alcohol-related complications

ICD-10: F10.1-F10.6 (alcohol dependence), Z72.1 (alcohol abuse)

*(definitions: [http://apps.who.int/classifications/icd10/browse/2010/en#/F10-F19](http://apps.who.int/classifications/icd10/browse/2010/en#/F10-F19).)*

AND/OR

Redemption of at least one prescription for disulfiram (ATC- code: N07BB01)

Liver cirrhosis

ICD-8: 571.09, 571.92, 571.99, 571.90, 571.91; ICD-10: K70.2, K70.3, K70.4, K74.3, K74.4, K74.5 K74.6

**B. Charlson Comorbidity Index and comorbidity groups.**

| **Charlson comorbidity category** | **ICD-8** | **ICD-10** | |
| --- | --- | --- | --- |
| Myocardial infarction | 410 | | I21;I22;I23 |
| Congestive heart failure | 427.09;427.10; 427.11;427.19; 428.99; 782.49 | | I50; I11.0; I13.0; I13.2 |
| Peripheral vascular disease | 440; 441; 442; 443; 444; 445 | | I70; I71; I72; I73; I74; I77 |
| Cerebrovascular disease | 430-438 | | I60-I69; G45; G46 |
| Dementia | 290.09-290.19; 293.09 | | F00-F03; F05.1; G30 |
| Chronic pulmonary disease | 490-493; 515-518 | | J40-J47; J60-J67; J68.4; J70.1;  J70.3; J84.1; J92.0; J96.1; J98.2; J98.3 |
| Connective tissue disease | 712; 716; 734; 446; 135.99 | | M05; M06; M08; M09;M30;M31; M32; M33; M34; M35; M36; D86 |
| Ulcer disease | 530.91; 530.98; 531-534 | | K22.1; K25-K28 |
| Mild liver disease | 571; 573.01; 573.04 | | B18; K70.0-K70.3; K70.9; K71; K73; K74; K76.0 |
| Diabetes type1    Diabetes type2 | 249.00;249.06; 249.07; 249.09  250.00;250.06; 250.07; 250.09 | | E10.0, E10.1; E10.9  E11.0; E11.1; E11.9 |
| Hemiplegia | 344 | | G81; G82 |
| Moderate to severe renal disease | 403; 404; 580-583;584;590.09; 593.19; 753.10-753.19; 792 | | I12; I13; N00-N05; N07; N11; N14; N17-N19; Q61 |
| Diabetes with end organ damage | 249.01-249.05; 249.08  250.01-250.05; 250.08 | | E10.2-E10.8  E11.2-E11.8 |
| Any tumor | 140-194 | | C00-C75 |
| Leukemia | 204-207 | | C91-C95 |
| Lymphoma | 200-203;275.59 | | C81-C85; C88; C90; C96 |
| Metastatic solid tumor | 195-198; 199 | | C76-C80 |
| AIDS | 079.83 | | B21-B24 |
